# Droplet Digital PCR Is a Novel Screening Method Identifying Potential Cardiac G-Protein-Coupled Receptors as Candidate Pharmacological Targets in a Rat Model of Pressure-Overload-Induced Cardiac Dysfunction

**DOI:** 10.3390/ijms241813826

**Published:** 2023-09-07

**Authors:** Nabil V. Sayour, Viktória É. Tóth, Regina N. Nagy, Imre Vörös, Tamás G. Gergely, Zsófia Onódi, Noémi Nagy, Csaba Bödör, Barnabás Váradi, Mihály Ruppert, Tamás Radovits, Federico Bleckwedel, Laura C. Zelarayán, Pal Pacher, Bence Ágg, Anikó Görbe, Péter Ferdinandy, Zoltán V. Varga

**Affiliations:** 1 Department of Pharmacology and Pharmacotherapy, Semmelweis University, 1085 Budapest, Hungary; sayour.nabil@gmail.com (N.V.S.);; 2 HCEMM-SU Cardiometabolic Immunology Research Group, 1085 Budapest, Hungary; 3 MTA-SE Momentum Cardio-Oncology and Cardioimmunology Research Group, Semmelweis University, 1085 Budapest, Hungary; 4 1st Department of Pathology and Experimental Cancer Research, Semmelweis University, 1085 Budapest, Hungary; 5 Heart and Vascular Center, Semmelweis University, 1085 Budapest, Hungary; 6 Institute of Pharmacology and Toxicology, University Medical Center Goettingen (UMG), 37075 Göttingen, Germany; 7 German Center for Cardiovascular Research (DZHK) Partner Site, 37075 Goettingen, Germany; 8 Laboratory of Cardiovascular Physiology and Tissue Injury, National Institute on Alcohol Abuse and Alcoholism, National Institute of Health, Rockville, MD 20852, USA; 9 MTA-SE System Pharmacology Research Group, Department of Pharmacology and Pharmacotherapy, Semmelweis University, 1085 Budapest, Hungary; 10 Pharmahungary Group, 6720 Szeged, Hungary

**Keywords:** heart failure, G-protein-coupled receptor, GPCR, prostaglandin F2α receptor, *Ptgfr*, cysteinyl leukotriene receptor 1, *Cysltr1*, drug repurposing, droplet digital PCR, ddPCR

## Abstract

The identification of novel drug targets is needed to improve the outcomes of heart failure (HF). G-protein-coupled receptors (GPCRs) represent the largest family of targets for already approved drugs, thus providing an opportunity for drug repurposing. Here, we aimed (i) to investigate the differential expressions of 288 cardiac GPCRs via droplet digital PCR (ddPCR) and bulk RNA sequencing (RNAseq) in a rat model of left ventricular pressure-overload; (ii) to compare RNAseq findings with those of ddPCR; and (iii) to screen and test for novel, translatable GPCR drug targets in HF. Male Wistar rats subjected to transverse aortic constriction (TAC, *n* = 5) showed significant systolic dysfunction vs. sham operated animals (SHAM, *n* = 5) via echocardiography. In TAC vs. SHAM hearts, RNAseq identified 69, and ddPCR identified 27 significantly differentially expressed GPCR mRNAs, 8 of which were identified using both methods, thus showing a correlation between the two methods. Of these, Prostaglandin-F2α-receptor (*Ptgfr*) was further investigated and localized on cardiomyocytes and fibroblasts in murine hearts via RNA-Scope. Antagonizing *Ptgfr* via AL-8810 reverted angiotensin-II-induced cardiomyocyte hypertrophy in vitro. In conclusion, using ddPCR as a novel screening method, we were able to identify GPCR targets in HF. We also show that the antagonism of *Ptgfr* could be a novel target in HF by alleviating cardiomyocyte hypertrophy.

## 1. Introduction

Despite the current advances in the pharmacotherapy for heart failure (HF), this disease remains a leading cause of mortality and hospitalization worldwide. Thus, the development of new therapeutic approaches is needed [1,2,3,4].

Studying novel pathomechanisms in HF is of paramount importance for identifying future drug targets; however, developing an entirely new drug from discovery to approval is time- and resource-consuming, and these maintain a low success rate, especially in the cardiovascular field [5,6]. Nevertheless, drug repurposing (also known as drug repositioning), i.e., the use of already approved drugs for novel indications, may decrease the risk, cost and time of the drug developmental process, as these drugs have already passed clinical phases I-II-III, and their safety has already been assessed [7]. For drug repositioning purposes, the family of G-protein-coupled receptors (GPCRs), representing the largest family of targets for already approved drugs, may offer adequate candidates for novel HF indications, since these are importantly involved in the physiology and pathophysiology of cardiovascular diseases [8].

There are GPCRs, such as ß-adrenoceptors and angiotensin-II receptor type 1, that are already targeted by drugs approved for HF treatment in clinical practice (i.e., by ß-blockers and angiotensin receptor blockers, respectively) [9]. In addition, several other GPCRs have been shown to play a pivotal role in HF in the preclinical models of HF, and therefore, are of promising translational value [10]. However, no systematic screening with predefined selection criteria for novel cardiac GPCR targets has been achieved to date in HF.

Despite the fact that GPCRs as drug targets are appealing, detecting their expression on a protein level (e.g., by Western blot or proteomics) is difficult and largely unsuccessful [11] because (i) GPCRs are insoluble integral membrane proteins that contain seven membrane-spanning helices, (ii) their relative expression is low, (iii) their protein dynamics and conformational diversity are currently often unresolved, and (iv) there is a lack of specific and high-affinity antibodies. Therefore, the transcriptomic analysis of GPCR expression is still a very useful approach to perform and can be used to screen for novel drug targets in the normal and diseased tissues. 

For transcriptomic profiling, bulk RNA sequencing is an unbiased, highly sensitive and high-throughput method, and, therefore, it became the gold standard choice for target screening on the level of gene expression. Nevertheless, sequence processing requires extensive bioinformatic resources to perform quality control, read mapping and differential gene expression analyses [12]. Droplet digital polymerase chain reaction (ddPCR), on the other hand, is a highly precise, absolute quantitative method for analyzing gene expression in a unit of measure of copies/μL, and does not require bioinformatic background to obtain comparable transcriptomic data [13]. Although ddPCR may not be feasible to analyze the whole transcriptome, when the transcriptional profiling is restricted to a family of targets, e.g., to GPCRs, ddPCR provides unbiased data.

Here, we hypothesized that the expression of cardiac GPCRs differs in conditions of cardiac dysfunction compared to physiological states, and that some of these differentially expressed cardiac GPCRs may be targets for already approved drugs. In addition, we hypothesized that beside bulk deep RNA sequencing, ddPCR is also a feasible method for unbiased screening within a family of targets, i.e., GPCRs. To this end, we aimed to investigate the differential expression of GPCR genes of pressure-overload-induced cardiac dysfunction vs. healthy rat hearts using bulk deep RNA sequencing and ddPCR. Then, we aimed to select cardiac GPCRs for further investigation based on the following criteria: (i) only those GPCRs that were found to be significantly differentially expressed via bulk deep RNA sequencing and via ddPCR; (ii) of these, GPCRs that show significant correlation in expression measured via bulk deep RNA sequencing and ddPCR; (iii) of these, GPCRs that have never been investigated in the context of cardiac dysfunction before and have commercially available pharmacological modulators. Finally, we aimed to test the efficacy of this/these drugs in an in vitro model of cardiomyocyte hypertrophy.

## 2. Results

### 2.1. Echocardiographic Characterization of Pressure-Overload-Induced Cardiac Dysfunction in Rats

**Figure 1:**
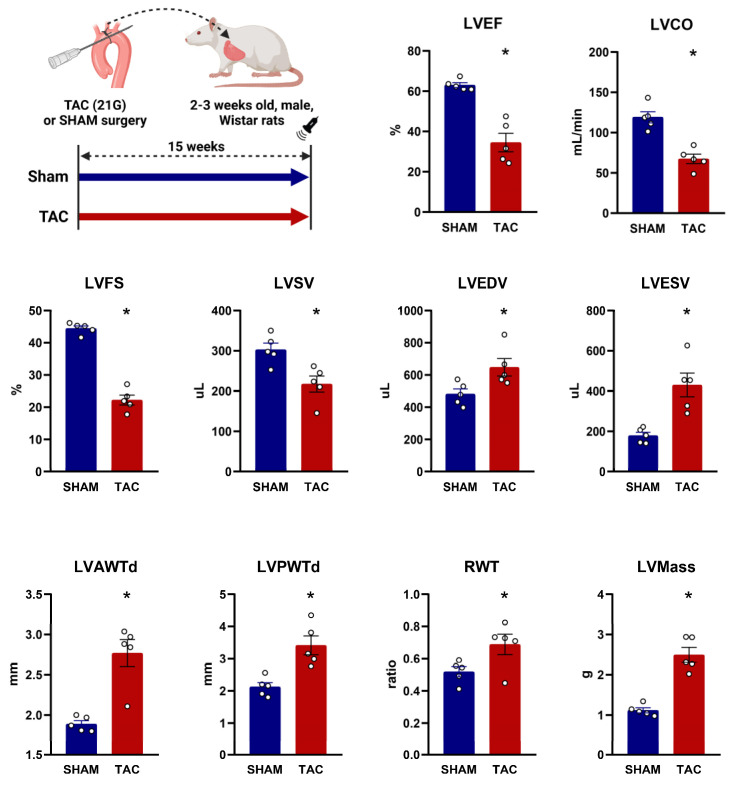
Echocardiographic parameters 15 weeks after transverse aortic constriction or sham surgery in rats. Left upper panel represents the experimental design. **TAC**: transverse aortic constriction; **LVEF**: left ventricular ejection fraction; **LVCO**: left ventricular cardiac output; **LVFS**: left ventricular fractional shortening; **LVSV**: left ventricular stroke volume; **LVEDV**: left ventricular end-diastolic volume; **LVESV**: left ventricular end-systolic volume; **LVAWTd**: left ventricular anterior wall thickness in diastole; **LVPWTd**: left ventricular posterior wall thickness in diastole; **RWT**: relative wall thickness; **LVMass**: calculated left ventricular mass. Measurements were performed on n = 5 individual animals for both the SHAM and the TAC groups. *: *p* < 0.05, unpaired Student’s *t*-test, shown as mean ± SEM.

**Figure 2:**
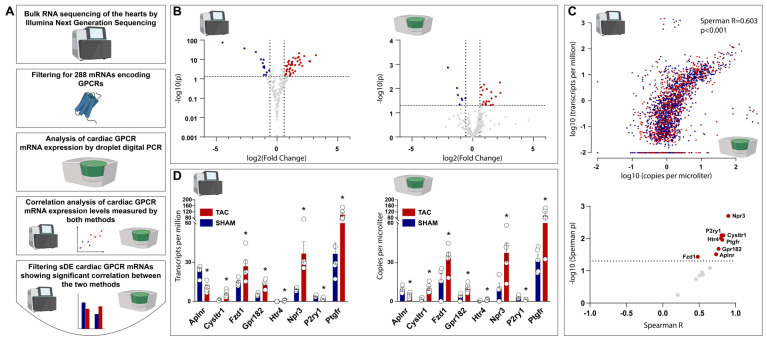
Transcriptional characterization of G–protein–coupled receptors in TAC vs. SHAM rat hearts by bulk RNA sequencing and droplet digital PCR. (**A**) represents the workflow for transcriptional characterization of cardiac G–protein–coupled receptors (GPCRs), followed by the comparison of results obtained from bulk RNA sequencing and droplet digital PCR (ddPCR), and by the selecting of GPCRs that were found using both methods to be significantly differentially expressed in a significant correlation. (**B**) volcano plots showing down– and up–regulation of cardiac GPCR expression measured via bulk RNA sequencing (left figure) and via ddPCR (right figure). (**C**) upper figure represents correlation between bulk RNA sequencing (Y axis) and ddPCR (X axis) values for gene expression, each dot represents one GPCR gene measured in one sample; lower figure represents GPCR genes that were found to be significantly differentially expressed (sDE) in TAC vs. SHAM rat hearts identified via both methods, only eight of which showed significant correlation between the two methods. (**D**) box plots showing those eight GPCR genes that were found to be sDE in TAC vs. SHAM rat hearts identified via both methods in significant correlation between the two methods. For each GPCR gene, measurements were obtained from n = 5 individual rat heart from both the SHAM and the TAC groups. For bulk RNA sequencing, *: *p* < 0.05 vs. SHAM, Wald test; for ddPCR, *: *p* < 0.05 vs. SHAM, Student’s unpaired *t*-test, shown as mean ± SEM.

To identify novel pharmacological targets for HF with a high possibility to be a candidate for drug repurposing, we aimed to investigate GPCR expression in pressure-overload-induced cardiac dysfunction vs. normal rat hearts. To this end, we used a widely established rat model by performing TAC surgery [14] and assessed cardiac function and morphology via terminal echocardiography (Figure 1). TAC surgery significantly reduced ejection fraction, cardiac output and fractional shortening compared to SHAM surgery at 15–18 weeks following operation. These findings were paralleled by a significant enlargement in cardiac dimensions, i.e., an increase in end-systolic and end-diastolic volumes and a significantly decreased stroke volume in the TAC group compared to the SHAM. Morphological analyses showed a significant increase in anterior, posterior and relative wall thicknesses of the heart, resulting in a significantly higher calculated left ventricular mass in the TAC vs. SHAM group (Figure 1). All in all, TAC surgery caused a severe phenotype of left ventricular pressure-overload-induced systolic dysfunction and cardiac hypertrophy, as expected [15]. After terminal echocardiography, cardiac samples were obtained from the left ventricles of both TAC vs. SHAM animals, and comparative transcriptional analyses were performed (Figure 2A).

### 2.2. Bulk RNA Sequencing Identified 69, and ddPCR Identified 27 Cardiac GPCR Genes to Be Differentially Expressed in TAC vs. SHAM Rat Hearts

After the completion of in vivo disease modeling, an unbiased whole transcriptome analysis on the cardiac samples of both TAC and SHAM hearts was performed via bulk RNA sequencing, resulting in a total of 5864 genes to be significantly differently expressed in TAC vs. SHAM hearts, after correcting for multiple comparisons (Supplementary Table S1 shows all significantly differentially expressed genes found via bulk RNA sequencing). Of note, several genes characteristic of the failing heart were found to be significantly differentially expressed in TAC vs. SHAM hearts, supporting echocardiographic findings [e.g., *nppa* (FC: 4.216, *p* < 0.001), *nppb* (FC: 1.799, *p* < 0.001), *atp2a2* (FC: −0.898, *p* < 0.001), *myh7* (FC: 1.424, *p* < 0.001), and several collagen genes].

The results of the bulk RNA sequencing were further filtered for a total of 288 GPCR genes (Figure 2A). Of these, 69 GPCR genes were found to be significantly differently expressed in TAC vs. SHAM hearts, 53 of which were up-regulated, and 16 were down-regulated (Figure 2B, left panel; Supplementary Table S2 shows all significantly differentially expressed GPCR genes measured via bulk RNA sequencing).

Parallel to the bulk RNA sequencing, we investigated the gene expression of a total of 288 GPCR genes by using ddPCR (Figure 2A). Of these GPCR genes, ddPCR found 27 genes to be significantly differentially expressed in TAC vs. SHAM hearts, 20 of which were up-regulated, and 7 of which were down-regulated (Figure 2B, right panel; Supplementary Table S3 shows all significantly differentially expressed GPCR genes measured via ddPCR). After the completion of the transcriptomic analyses, we aimed to compare the cardiac GPCR gene expression profiles measured via bulk RNA sequencing and ddPCR.

### 2.3. Comparative Analysis of Cardiac GPCR Gene Expression Profiles Measured via Bulk RNA Sequencing and ddPCR Shows Significant Correlation 

To compare how GPCR gene expression profiles may differ between bulk RNA sequencing and ddPCR, we performed a correlation between the TPM values (obtained from bulk RNA sequencing data) and the copies/μL values (obtained from ddPCR data) of all the 288 cardiac GPCR genes investigated from both the TAC and SHAM animals. The overall results of both methods showed significant correlation (Figure 2C, upper figure, Spearman R = 0.603, *p* < 0.001).

To avoid sources of selection bias for identifying possible GPCR targets in HF, we aimed to focus on GPCR genes that were found to be significantly differentially expressed in TAC vs. SHAM hearts both via bulk RNA sequencing, and via ddPCR, and show a significant correlation between the two methods (Figure 2A). A total of 14 cardiac GPCR genes were identified via both methods to be significantly differentially expressed with similar direction of expression changes (up-, or down-regulated) in TAC vs. SHAM hearts, 8 of which genes showed a significant correlation (Figure 2C, lower figure; Supplementary Figure S1 shows correlation analyses for each sDE GPCRs identified via the two methods). Individual values for gene expression levels of these cardiac GPCR genes are shown on Figure 2D. (left figure for bulk RNA sequencing, right figure for ddPCR).

### 2.4. Filtering for Novel GPCR Targets Identifies Prostaglandin F2α Receptor to Be a Potential GPCR Target with Relevant Clinical Translatability in Heart Failure

Of the eight cardiac GPCR genes that were identified to be significantly differentially expressed in TAC vs. SHAM hearts via both methods and a significant correlation, we sought genes that (i) can be targeted using commercially available small molecules, and (ii) have not been described yet in the context of HF.

Previous investigations have demonstrated that the inhibition of *Npr3* improves HF after myocardial infarction in mice [16], and that *Htr4* was involved in the pathogenesis of ischemic HF in rats [17]. However, to the best of our knowledge, *Ptgfr* was demonstrated to be involved in atherosclerosis and blood pressure regulation [18,19], bleomycin-induced pulmonary fibrosis [20], as well as in the collagen synthesis of cardiac fibroblasts [21], but *Ptgfr* has never been investigated in the pathogenesis of systolic dysfunction to date.

All in all, *Ptgfr* was chosen to be the candidate for further analyses, as this GPCR gene (i) showed a significant increase in TAC vs. SHAM hearts both via bulk RNA sequencing and ddPCR, with a significant correlation between the two methods (Spearman R = 0.817, *p* = 0.011, Supplementary Figure S1); (ii) has a commercially available antagonist (AL-8810); and (iii) has not been described yet in the context of HF.

In addition, given that *Cysltr1* also fits most of the above-stated criteria (as (i) the expression profile of *Cysltr1* also significantly correlates between bulk RNA sequencing and ddPCR, and (ii) *Cysltr1* has already-approved antagonists that are currently indicated in the maintenance treatment of asthma), it was previously brought in touch with HF [22], *Cysltr1* was also further characterized, the results of which are shown in the Supplementary Materials.

### 2.5. Ptgfr Is Expressed by Cardiac Fibroblasts and Cardiomyocytes

**Figure 3:**
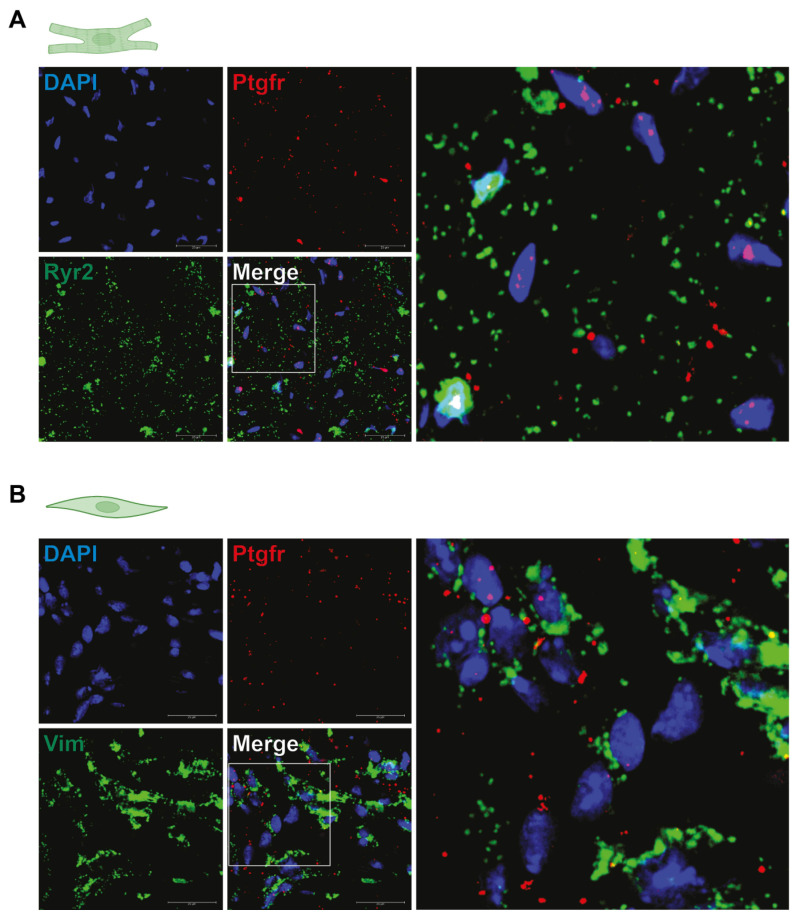
Prostaglandin F_2α_ receptor expression profile in murine hearts. (**A**,**B**) demonstrated representative RNA-Scope images of murine hearts showing *Ptgfr* expression on cardiomyocytes (Ryr2^+^ cells) and cardiac fibroblasts (Vim^+^ cells), respectively, with no technical replication or quantification.

First, we aimed to characterize which cell types of the cardiac tissue expresses *Ptgfr*. To this end, we performed RNAScope in situ hybridization analyses on the cardiac tissue of mice. We sought for co-localization of *Ptgfr* mRNA with markers of cardiomyocytes (RYR2), endothelial cells (CD31), fibroblast cells (VIM), smooth muscle cells (TAGLN), or macrophages (CD68). We found that *Ptgfr* gene was primarily expressed on RYR2+ cardiomyocytes and VIM+ fibroblast cells (Figure 3A,B, respectively, Supplementary Figure S2 shows representative RNA Scope images of *Ptgfr* expression in CD31+, TAGLN+ and CD68+ cardiac cells). Our findings showed similar results to those of the Tabula Muris open dataset for single cell transcriptomic data [23].

On the other hand, *Cysltr1* shows an expression profile that is not specific to certain cardiac cells, but is rather globally expressed, as measured by Tabula Muris [23]. Nevertheless, single cell RNAseq of mouse hearts showed that *Cysltr1* is predominantly expressed on macrophages [24] (Supplementary Figure S3A). Representative RNA Scope images show an expression of *Cysltr1* on RYR2+ cardiomyocytes and VIM+ fibroblast cells, as well as in endothelial cell populations (Supplementary Figure S3B–F).

### 2.6. Ptgfr Inhibition by AL-8810 Reverts Angiotensin-II Induced Hypertrophy of Neonatal Rat Cardiomyocytes

**Figure 4:**
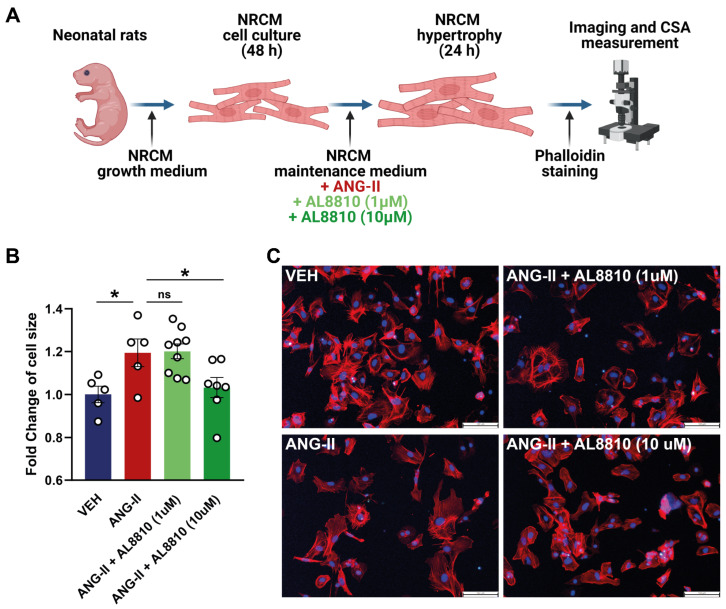
Effect of Prostaglandin F_2α_ receptor inhibition on in vitro angiotensin-II-induced cardiomyocyte hypertrophy. (**A**) represents the experimental workflow of neonatal rat cardiomyocyte (NRCM) cell culturing, induction of hypertrophy by angiotensin-II (ANG-II), treatment with prostaglandin F_2α_ receptor (*Ptgfr*) antagonist AL8810 in lower (1 μM) and higher (10 μM) doses, and measurement of cell surface area (CSA) after phalloidin staining. (**B**) shows significant increase in CSA of NRCM cells by ANG-II, which could be reverted by 10 μM of AL8810; biological replicates of *n* = 5–9/group, *: *p* < 0.05 vs. ANG-II, One-way ANOVA, Dunnett’s post hoc test, shown as mean ± SEM; ns: not significant. (**C**) shows representative images of phalloidin staining for each treatment groups.

Cardiomyocytes were identified to express *Ptgfr* in murines, as shown above. As the TAC model of HF is characterized by pressure-overload-induced cardiac hypertrophy, we aimed to test the effect of *Ptgfr*-inhibition in an in vitro model that recapitulates these characteristics of TAC model. To this end, we used an in vitro model of ANG-II-induced hypertrophy of neonatal rat cardiomyocytes, as described previously [25] (Figure 4A). In this model, we tested the effect of AL-8810, a highly selective *Ptgfr* inhibitor of lower (1 μM) or higher (10 μM) concentrations. These concentrations were selected based on previous publications using AL-8810 on in vitro cell cultures [21,26,27,28].

We found that ANG-II treatment resulted in a significant, ~1.25-fold increase in cell surface area of NRCM cells compared to the vehicle treated group (Figure 4B,C). Cardiomyocyte hypertrophy could be reverted by the 10 μM, but not the 1 μM concentrations of AL-8810 treatment (Figure 4B,C). This is the first demonstration of the anti-hypertrophic effect of *Ptgfr*-inhibition on cardiomyocytes.

In the same experimental setup, the effect of montelukast (Monte), a selective *Cysltr1* inhibitor of lower (1 μM) or higher (10 μM) concentrations showed no anti-hypertrophic effect (Supplementary Figure S4).

## 3. Discussion

Here, we demonstrate for the first time that *Ptgfr*, a cardiac GPCR is a potential pharmacological target in HF showing a significantly increased expression in TAC vs. SHAM hearts of rats. *Ptgfr* was identified as a result of a systematic screening for cardiac GPCR genes to be differentially expressed in TAC vs. SHAM rat hearts using bulk deep RNA sequencing, as well as ddPCR, a method that—to the best of our knowledge—was used for the first time for target screening purposes (Graphical abstract). In line with the literature, *Ptgfr* was demonstrated to be expressed in cardiac muscle cells and fibroblasts. Finally, here, we show that inhibition of *Ptgfr* by AL-8810 prevents in vitro cardiomyocyte hypertrophy induced by Ang-II, also supporting that *Ptgfr* is a potential pharmacological target in HF.

To enable the identification of drugs that are possible candidates of drug repurposing for a novel indication in HF, we aimed to identify GPCRs that show significantly different expression in TAC vs. SHAM hearts, as GPCRs are the largest family of targets for already-approved drugs—agonists, antagonists or partial agonists—currently available on the market. Given the general need for increasing translational value and success of preclinical studies in the field of cardiology [29,30,31,32,33], we aimed to conduct a systematic screening of cardiac GPCRs with reduced risk of subjective bias. To this end, we first assessed the cardiac GPCR gene expression both by bulk RNA sequencing and ddPCR, and filtered only for those candidates that (i) were identified by both methods to be significantly differentially expressed in TAC vs. SHAM rat hearts, and (ii) show significant correlation in levels of gene expression measured by both methods. Only 8 of the total of 288 GPCRs met the above criteria, majority of which have already been tested in previous studies of cardiovascular diseases, a fact that supports the validity of the currently used target screening approach.

Of these GPCRs, we aimed to further characterize *Ptgfr*, as this GPCR (i) shows a significantly increased expression level in failing vs. normal rat and human hearts, as also shown by the single-nucleus transcriptomic data of human failing hearts from patients with dilated or hypertrophic cardiomyopathy [34] (available through the Broad Institute’s Single Cell Portal under project ID SCP1303), a finding that supports potential translatability into the clinical settings, (ii) has a high abundance in the cardiac tissue, and especially on cardiac ventricular fibroblast and cardiomyocytes (iii) can be targeted by a highly selective commercially available antagonist, AL-8810, and (iv) is novel to be described in the context of HF and cardiomyocyte hypertrophy. Previous publications also evidence that prostaglandin-F2α (PGF2α)-*Ptgfr* axis may play a pivotal role in cardiovascular diseases [35,36]. An early study by Rabinowitz and colleagues described that PGF2α is mainly produced by cardiac fibroblasts, which increases via myocardial ischemia [37]. Later, it has been demonstrated that PGF2α further promotes PGF2α formation through a feed-forward fashion, which enhances fibrosis [20,38,39].

Beside the role of PGF2α-*Ptgfr* axis in fibrosis, Lai et al. demonstrated that in vitro treatment of NRCM with PGF2α results in cardiomyocyte hypertrophy comparable to that induced by phenylephrine and endothelin-1. This finding was paralleled by a ten-fold increase in the production of atrial natriuretic peptide, and was specific only to PGF2α amongst all investigated prostanoids (i.e., PGA2, PGB2, PGD2, PGF2α, PGI2, carbaprostacyclin and a thromboxane analogue), as other prostanoids could not cause hypertrophy [40]. Moreover, a seminal study by Yu and colleagues showed that the deletion or disruption of *Ptgfr* (i) leads to reduced blood pressure, (ii) lowers the activity of the renin–angiotensin–aldosterone–system, and, perhaps most importantly, (iii) attenuates atherogenesis in LDLR−/−mice [18].

Regarding the relationship between PGF2α-*Ptgfr* axis and HF, Mallat et al. showed that 8-iso-PGF2α is significantly elevated in pericardial fluid of patients with HF, the level of which significantly and positively correlates with HF severity [41]. In the current study, we demonstrate that the expression of *Ptgfr* shows a significant change in TAC vs. SHAM rat hearts, and that inhibition of this GPCR prevents Ang-II-induced cardiomyocyte hypertrophy in vitro. This finding is in alignment with previous publications, emphasizing that the inhibition of *Ptgfr* could be beneficial in patients with cardiovascular disease, and more specifically, with HF, as discussed above.

Other GPCRs found by the current screening approach to be potential targets in HF were also touched upon by previous studies. For instance, the expression of *Npr3*, a receptor that is believed to be involved in natriuretic peptide clearance, was found to be significantly elevated in failing rat and human hearts [42,43]. The inhibition of Npr3 by osteocrin (either by exogenous administration, or in osteocrin-transgenic mice) results in a significantly increased survival of mice undergoing myocardial infarction induced chronic HF, accompanied by improved cardiac function, lesser degree of cardiac inflammatory processes, and smaller ischemic area [16]. In another set of experiments using isoproterenol-induced in vivo model of HF, intramyocardial administration of an *Npr3* siRNA reduced cardiac hypertrophy and fibrosis, and elevated the levels of circulating A-type natriuretic peptide [44]. In addition, microRNAs (more precisely, miR-100 and miR-143) were also identified to be novel candidates for treating HF through interacting with Npr3 expression [45,46].

*Aplnr*, a GPCR that shares remarkable homology with the angiotensin-II type 1 receptor, was found to be significantly down-regulated in failing vs. normal rat hearts in the current experiments. Activation of *Aplnr* by elabela was shown to revert TAC-induced HF and Ang-II-induced hypertension in mice [47]. In addition, treatment with an *Aplnr* agonist reduced myocardial infarct size, and increased serum nitric oxide level in rats [48]. Of interest, a complete loss of apelin—an endogenous ligand of Aplnr—results in increased mortality of mice with myocardial infarction, paralleled by increase in infarct size and inflammation [49].

In the current in vivo experiments, we found that level of *Cysltr1* expression is significantly increased in TAC vs. SHAM rat hearts, a finding that is also partially supported by the data derived from single nucleus RNA sequencing of human hearts showing a tendentious—but not significant—elevation in failing vs. normal cardiac tissues [34]. Remarkably, antagonists of *Cysltr1*, i.e., montelukast or zafirlukast are currently available on the market indicated for the maintenance treatment of asthma, a fact showing that antagonists of *Cysltr1* are potential candidates of drug repurposing for a novel indication in HF, which is also supported by other clinical and preclinical studies. For instance, Hoxha and colleagues demonstrated that the use of montelukast in asthmatic patients significantly decreases the incidence of cardiovascular events in a propensity-score matched study, compared to non-users [50]. This is in parallel to the finding of Ingelsson et al., also showing that montelukast may have a potential role for secondary prevention of cardiovascular disease [51]. Moreover, a recent preclinical study demonstrated that montelukast improves cardiac function in a dose-dependent manner in a mouse model of TAC-induced HF [22]. Overall, the screening approach established in the current study was able to identify *Cysltr1* as a highly potential pharmacological target, antagonist of which is a candidate of drug repurposing for HF.

Other cardiac GPCRs with significantly different expression in TAC vs. SHAM rat hearts identified only by ddPCR in the current study were also shown to be involved in the pathogenesis of HF. For instance, the pituitary adenylate cyclase-activating polypeptide (PACAP) receptor type 1 (*Adcyp1r1*) was previously shown to mediate the detrimental effects of PACAP, a neuropeptide in HF of different etiologies [52,53]. Endothelin and its receptors (*Ednra*, *Ednrb*) were also previously associated with HF; however, inhibition of endothelin receptors by bosentan failed to improve outcomes of patients with severe chronic HF [54]. Antagonism of glucagon receptor (*Gcgr*), a GPCR showing significantly lower expression in TAC vs. SHAM rat hearts, was previously demonstrated to improve cardiac function of animals with HF of ischemic and non-ischemic etiologies [55]. A detailed in silico analysis on failing and healthy human hearts has demonstrated that cardiac GPCRs encoded by the *F2rl2* and *Sstr5* genes could serve as novel and potential biomarkers or therapeutic targets of HF [56], similarly to our findings identified by ddPCR. Since, to the best of our knowledge, the remaining cardiac GPCRs identified by the current screening approach using ddPCR (Supplementary Table S3) were not yet brought in context with the pathophysiology of HF, future studies investigating the role of these GPCRs in HF would be of great interest.

Despite promising results, there are several limitations to the currently used screening approach. We assessed the level of GPCR gene expression, because GPCRs are difficult and often unsuccessful to be detected at a protein level (either by Western blot, immunohistochemistry, or by proteomics). This approach may be confounded by the lack of information regarding post-translational processes of GPCRs; however,, transcriptomic measurements of GPCR expression are widely used and in the present work allowed for systematic screening of a total of 288 GPCRs. Another important characteristic of the current screening approach could be that GPCRs showing lower absolute expression levels may not be measured by ddPCR with high reliability. This is demonstrated by the pattern of the correlation analysis between the gold standard bulk RNA sequencing and ddPCR showing that GPCR genes with lower expression levels (i.e., TPM and copies/uL < 1) could be more differentiated by bulk RNA sequencing than that by ddPCR. On the other hand, however, GPCR genes with higher expression levels (i.e., TPM and copies/uL > 1) could be differentiated with both methods similarly, and overall, the correlation was significant and meaningful between the two methods. Finally, although promising, these results regarding *Ptgfr*, and *Ptgfr*-inhibition should be validated in a variety of in vitro and in vivo studies. For instance, on-target and off-target effects of AL-8810, and other *ptgfr*-antagonists (as well as agonists) in similar cell culture studies deserve further investigations in the future. Furthermore, beside the TAC model of HF, *Ptgfr*-modulation should be tested in models of myocardial infarction-induced-HF, Ang-II or isoprenaline-induced cardiac damage, or aorto-ventricular-fistula-induced HF in different species. Moreover, where possible, the influence of sex and age should also be taken into account in future in vivo studies.

## 4. Materials and Methods

### 4.1. Ethical Approval

This investigation complies with the Guide for the Care and Use of Laboratory Animals published by the US National Institutes of Health (NIH Publication No. 85–23, revised 1996), and with the guidelines from Directive 2010/63/EU of the European Parliament on the protection of animals used for scientific purposes. Investigations were compliant with local directives and approved by The Animal Ethics Committees at Semmelweis University, Budapest (PE/EA/1784-7/2017, and PEI/001/2374-4/2015). This study was conducted in accordance with the ARRIVE guidelines. 

### 4.2. Rat Model of Transverse Aortic Constriction Induced Cardiac Dysfunction and Hypertrophy

Animals were housed under standard conditions (25 ± 2 °C and constant 12 h light/dark) and allowed free access to a standard rodent chow diet and tap water ad libitum. Prior to the interventions, at least 7 days of acclimatization period was applied. For all in vivo studies, animals were randomly assigned to experimental groups, and data analyses were performed by 1–3 independent persons in a blinded fashion. Animals were excluded from further analyses if iatrogenic death or severe complications occurred during or after surgery (e.g., excessive bleeding, acute HF). During the surgery, body temperature was continuously monitored and maintained at 37 °C. Pain reflex was monitored by pinching the toes every 5–10 min.

Male Wistar rats (2–3 weeks old, 50–75 g, purchased from Toxi-Coop Zrt., Dunakeszi, Hungary) were randomly assigned to sham (SHAM) or transverse aortic constriction (TAC) surgery. Under isoflurane anesthesia (5 *V*/*V*% isoflurane for induction and 1.5–2 *V*/*V*% isoflurane for maintenance after orotracheal intubation, 100% O_2_), after removal of the chest hair and disinfecting the surgical area, a left anterolateral thoracotomy was performed in the 2nd intercostal space under surgical stereomicroscope. After the partial removal of the thymus, the aortic arch between the brachiocephalic trunk and the left common carotid artery was identified by atraumatically dissecting the surrounding connective tissue. The aorta was constricted to the external size of a 21-gauge needle in the TAC animals. SHAM animals underwent the same procedure without the completion of the aortic constriction. Thereafter, thorax was closed by suturing the 2nd and 3rd ribs followed by the suturing of the skin. To prevent postoperative pain and dehydration, tramadol and physiological saline (10 mg/kg in 0.5 mL) was injected subcutaneously before the animals regained consciousness. After a median follow-up of 15 weeks, echocardiography was performed and animals were euthanized by terminal arterial blood collection under deep anesthesia, followed by cardiac sample collection for further analyses.

### 4.3. Echocardiography

An echocardiographic imaging unit (Vivid i; GE Healthcare, Waukesha, WI, USA) with a 13-MHz linear probe (GE 12L-RS; GE Healthcare) at a constant frame rate of 218 frames/s was used for echocardiographic measurements. Animals were anesthetized by isoflurane (5 *V*/*V*% isoflurane for induction and 1.5–2 *V*/*V*% isoflurane for maintenance through a nose cone, 100% O_2_) and were placed onto a heating pad in a supine position to maintain body temperature at 37 °C. Chest hair was removed to obtain an optimal acoustic window. Echocardiographic cines were taken in 2D parasternal long-axis (PLAX) and short-axis (PSAX) views. Left ventricular end-systolic and end-diastolic volumes were derived from the rotational volumes of the left ventricular trace at diastole and systole, around the long axis line of the spline (LVESV, LVEDV, respectively), obtained from the PLAX view in B-mode acquisition. Left ventricular stroke volume (LVSV) was calculated as LVEDV-LVESV. Left ventricular ejection fraction (LVEF) was calculated as [LVSV/LVEDV] * 100. Left ventricular cardiac output (LVCO) was calculated as [heart rate * LVSV]/1000. Left ventricular end-systolic and end-diastolic diameters (LVESD and LVEDD, respectively), as well as left ventricular anterior and posterior wall thicknesses in diastole (LVAWTd and LVPWTd, respectively) were obtained from the PSAX view in M-mode acquisition at midpapillary level. Relative wall thickness (RWT) was calculated as 2 * LVPWTd/LVEDD. LVMass was calculated according to the modified cubic formula as 1.04 * {[(LVEDD  +  LVAWd  +  LVPWd) * 3  −  LVIDd * 3] * 0.8  +  0.6}. Cines were obtained and analyzed (EchoPAC; GE Healthcare) in a blinded fashion by a single operator.

### 4.4. RNA Isolation

Total RNA was isolated from rat hearts by using a chloroform/isopropanol precipitation method. Briefly, Qiazol^®^ (Qiagen, Venlo, The Netherlands) was added to each sample and homogenized with TissueLyser (Qiagen, Venlo, The Netherlands). Homogenates were then centrifuged, and from the clean upper phase, DNA and protein were precipitated with chloroform, followed by precipitation of the total RNA using isopropanol. Pellets were washed four times with 75% ethanol (vWR, Radnor, PA, USA), then total RNA was resuspended in nuclease-free water. Finally, RNA concentrations for each sample were determined by spectrophotometry (Implen Nanophotometer^®^ N60, München, Germany).

### 4.5. RNA Sequencing and Bioinformatic Analysis

The RNA Integrity Numbers and RNA concentration were determined by RNA ScreenTape system with 2200 Tapestation (Agilent Technologies, Santa Clara, CA, USA) and RNA HS Assay Kit with Qubit 3.0 Fluorometer (Thermo Fisher Scientific, Waltham, MA, USA).

For mRNA-Seq library construction, NEXTFLEX^®^ Rapid Directional RNA-Seq Kit 2.0 with Poly(A) Beads 2.0 (PerkinElmer, Waltham, MA, USA) was applied according to the manufacturer’s protocol. The quality and quantity of the library was determined by using High Sensitivity DNA1000 ScreenTape system with 2200 Tapestation (Agilent Technologies, Santa Clara, CA, USA) and dsDNA HS Assay Kit with Qubit 3.0 Fluorometer (Thermo Fisher Scientific, Waltham, MA, USA). Pooled libraries were diluted to 1.6 pM for 2 × 80 bp paired-end sequencing with 150-cycle High Output v2 Kit on the NextSeq 500 Sequencing System (Illumina, San Diego, CA, USA) according to the manufacturer’s protocol. 

During preprocessing of raw sequencing data by Cutadapt (version 3.0) adapter sequences, poly(A) tails and bases with a Phred score below 30 were trimmed, reads below a length of 19 nt were filtered out [57,58]. Quality of reads was checked by FastQC (version 0.11.8) and MultiQC (version 1.7) software [59]. Alignment and annotation of reads were performed by Hisat2 (version 2.0.4) and featureCounts (version 2.0.0), respectively using Ensembl Rnor 6.0. reference genome and annotation [60,61]. Sequence alignment map (SAM) files were converted to binary form by Samtools (version 1.9) [62,63]. Differential expression analysis and calculation of transcripts per million (TPM) was conducted in R environment (version 3.2.3) with the usage of DESeq2 (version 1.10.1) package [64]. *p* values of Wald tests were adjusted by Benjamini-Hochberg method due to multiple comparisons [65]. After the completion of whole transcriptome sequencing and bioinformatic analysis, data was screened for GPCRs.

### 4.6. Droplet Digital PCR

Screening and quantitative assessment of cardiac GPCR expression were performed by droplet digital polymerase chain reaction (QX200 Droplet Digital PCR System; Bio-Rad Laboratories, Hercules, CA, USA) using a pre-designed assay kit allowing for the measurement of 288 GPCRs (PrimePCR Pathway Plate, 96 well; GPCR Tier 1-2-3 R96, Rat; Bio-Rad Laboratories, Hercules, CA, USA). Absolute quantification of the target molecules was performed using water-oil emulsion droplet technology. Briefly, each sample was fractionated into 15–20.000 droplets by QX200 Droplet Generator (DG32 Automated Droplet Generator Cartridges, Automated Droplet Generation Oil for EvaGreen; Bio-Rad Laboratories, Hercules, CA, USA), and PCR amplification of the template molecules occurs in each individual droplet. Detection of gene expression measured by QX Droplet Reader System using ddPCR Droplet Reader Oil (Bio-Rad Laboratories, Hercules, CA, USA). cDNA was synthesized from 4 μg total RNA by iScript Advanced cDNA Synthesis Kit (Bio-Rad Laboratories, Hercules, CA, USA) according to the manufacturer’s protocol. cDNA was further diluted 20× with RNAse free water. All reactions were carried out using QX200 ddPCR EvaGreen Supermix (Bio-Rad Laboratories, Hercules, CA, USA) and 100 ng of input cDNA. Level of GPCR gene expression, i.e., copies/μL was quantified by QuantaSoftTM Analysis Pro (version 1.0.596, Bio-Rad Laboratories, Hercules, CA, USA). 

### 4.7. Neonatal Rat Cardiomyocyte Model of Hypertrophy 

In vitro model of cardiomyocyte hypertrophy was performed as described earlier [25,66]. Briefly, primary neonatal rat cardiomyocytes (NRCM) were isolated from neonatal rats of both sexes (post-partum days 1–2). After disinfection by 70% ethanol, animals were euthanized by cervical dislocation, followed by the excision of the hearts, which were then transferred into phosphate-buffered saline (PBS, pH = 7.2). Then, ventricles were separated and gently minced by using fine forceps, followed by a digestion in 0.25% trypsin solution (5 mL per heart) at 37 °C for 90 min. Cell suspension was then centrifuged at 300 g at 4 °C for 15 min, supernatant was discarded, and pellets were resuspended in growth medium (glucose and glutamine-rich Dulbecco’s MEM [10-014-CV, Corning Inc., Corning, NY, USA] supplemented with 10% fetal bovine serum, 1% L-glutamine [25030081, Life Technologies Corporation, Carlsbad, CA, USA] and 1% antibiotic/antimycotic solution [30004CI, Corning Inc., New York, NY, USA]) and plated onto 6-well plates (1.0–1.2 × 10^6^ cells/well, in 2 mL growth medium) to eliminate fibroblasts at 37 °C for 25 min (pre-plating step). Cells of the supernatant were then re-plated onto fresh 24-well plates onto coverslips (1.0–1.2 × 10^5^ cells/well, in 1 mL growth medium), and were kept in 5% CO_2_ incubator at 37 °C. On the following day, medium was changed to a fresh medium.

To achieve hypertrophy of NRCM cells, medium was changed again to a fresh medium at the second day after the isolation, and cells were treated with 1 μM of angiotensin-II (ANG-II; A9525, Sigma, St. Louis, MO, USA) in DMSO, as vehicle. For control treatment, cells received DMSO only. ANG-II treated cells received either no additional treatment, or AL-8810 (a selective inhibitor for *Ptgfr*; A3846, Sigma, St. Louis, MO, USA) or montelukast (Monte; a selective inhibitor of *Cysltr1*; GX0261, Glentham Life Sciences Ltd., Corsham, UK) in DMSO at the concentrations of 1 μM or 10 μM. The volume of the vehicle was equal in all the groups. 24 h after start of treatment, cells were fixed with 2% paraformaldehyde in 1 × PBS for 5 min at room temperature, then permeabilized with 0.2% Triton-X (Sigma, St. Louis, MO, USA) for 10 min. Slides were then stained with phalloidin iFluor-594 (ab176757, Abcam, Cambridge, UK) and DAPI, and images were taken by Leica LMD6 microscope. The surface area of at least 150 cells from 6 independent and random fields was measured by two blinded and independent experimenters using the ImageJ software (version 1.8.0). The average cell surface area of all measured cells in a treatment group was used as one data point, and each datapoint represents one biological replicate. We pre-defined the following exclusion criteria: (i) if the ANG-II treatment increased the cell surface area by <5%, and (ii) if cell viability was <90%.

### 4.8. RNA Scope^®^ In Situ Hybridization Assay

The in situ hybridization assay was performed on the cross section slides of the ventricles harvested from mouse heart samples using RNA Scope^®^ Multiplex Fluorescent Kit v2 according to the manufacturer’s instructions (Advanced Cell Diagnostics Pharma Assay Services, Newark, CA, USA). Briefly, formalin-fixed paraffin-embedded tissue sections were baked for 1 h at 60 °C, and then deparaffinized. Endogenous HRP activity was blocked with hydrogen peroxide (catalog number: 322335) treatment for 10 min at room temperature. Target retrieval was performed for 15 min at 100 °C, followed by Protease Plus (catalog number: 322331) treatment for 15 min at 40 °C. Probes were then hybridized for 2 h at 40 °C (3-plex Positive Control Probe mm (catalog number: 320881), 3-plex Negative Control Probe (catalog number: 320871), Mm-*Ptgfr*-O1 (catalog number: 501841, accession no.: NM_008966.3), Mm-*Cysltr1*-C3 (catalog number: 487541-C3, accession no.: NM_021476.5), Mm-Vim-C2 (catalog number: 457961-C2, accession no.: NM_011701.4), Mm-Cd68 (catalog number: 316611, accession no.: NM_009853.1), Mm-Cd68-C3 (catalog number: 316611-C3, accession no.: NM_009853.1), Mm-Pecam1-C2 (catalog number: 316721-C2, accession no.: NM_001032378.1), Mm-Ryr2-C2 (catalog number: 479981-C2, accession no.: NM_023868.2), and Mm-Tagln-C2 (catalog number: 480331-C2, accession no.: NM_011526.5)). Cell type-specific markers were used to identify cardiomyocytes with a probe recognizing the mRNA of Ryanodine receptor 2 (RYR2) [67], endothelial cells with a probe recognizing the mRNA of platelet endothelial cell adhesion molecule 1 (PECAM-1, also known as CD31) [68], fibroblast cells with a probe recognizing the mRNA of Vimentin (VIM) [69,70], smooth muscle cells with a probe recognizing the mRNA of transgelin (TAGLN) [71], and macrophages with a probe recognizing the mRNA of cluster of differentiation 68 (CD68) [72], respectively. Afterwards RNA Scope amplification was performed followed by signal development with TSA fluorophores (TSA-Cy3, TSA-FITC, Akoya Biosciences, Marlborough, MA, United States). Nuclei were counterstained with DAPI (catalog number: 323108) and mounted with Prolong Gold Antifade Reagent (catalog number: 9071S, Cell Signaling Technology, Danvers, MA, USA). Specific RNA staining signal was identified as red/green dots. Fluorescent signals were detected using a Leica DMI8 Confocal microscope (Leica, Wetzlar, Germany).

### 4.9. Statistical Analysis

All data were generated from at least four independent experiments. All values are presented as mean ± standard error of the mean (SEM). Statistical analysis was performed using GraphPad Prism (version 8.0.1). *p* < 0.05 was considered significant. Normal distribution of data was tested by Shapiro–Wilk normality test. For comparisons between two groups, either parametric two-tailed Student’s *t*-test, or nonparametric Mann–Whitney U-test was performed. For comparison of multiple groups to one control group, one-way ANOVA followed by Dunnett’s post hoc test was used. The post hoc tests were conducted only if F in ANOVA test achieved *p*  <  0.05 and there was no significant variance in homogeneity. For correlation analysis of two continuous variables, Spearman’s rho (R) was computed. ROUT analysis was performed to identify outliers, with Q value  =  1%.

## 5. Conclusions

Here, we established a systematic screening approach by using ddPCR in comparison to bulk RNA sequencing data, and found that cardiac GPCR expression differs in hearts with pressure-overload-induced cardiac dysfunction vs. healthy hearts in a rat model. In addition, we demonstrated that some of these GPCRs are targets for already-approved drugs that are available commercially. Here, we show, for the first time, that *Ptgfr* expression is significantly increased in TAC vs. SHAM rat hearts, a GPCR that is primarily expressed by cardiac fibroblast and cardiomyocytes, and that the inhibition of *Ptgfr* by the small molecule AL 8810 prevents Ang-II-induced cardiomyocyte hypertrophy in vitro. In addition, the current screening approach was also able to identify other cardiac GPCRs that have already been investigated in the context of HF, and that antagonists of *Cysltr1*, i.e., montelukast or zafirlukast could be potential candidates of drug repurposing for a novel indication in HF, which deserves further investigation.

## Data Availability

The datasets used and/or analyzed are available from the corresponding author upon request. The RNA sequencing datasets will be deposited in the ArrayExpress database.

## Supplementary Materials

The supporting information can be downloaded at: https://www.mdpi.com/article/10.3390/ijms241813826/s1.

## References

[1] Murphy S.P., Ibrahim N.E., Januzzi J.L. (2020). Heart Failure with Reduced Ejection Fraction. JAMA.

[2] Shah K.S., Xu H., Matsouaka R.A., Bhatt D.L., Heidenreich P.A., Hernandez A.F., Devore A.D., Yancy C.W., Fonarow G.C. (2017). Heart Failure with Preserved, Borderline, and Reduced Ejection Fraction. J. Am. Coll. Cardiol..

[3] Vergaro G., Ghionzoli N., Innocenti L., Taddei C., Giannoni A., Valleggi A., Borrelli C., Senni M., Passino C., Emdin M. (2019). Noncardiac Versus Cardiac Mortality in Heart Failure with Preserved, Midrange, and Reduced Ejection Fraction. J. Am. Heart Assoc..

[4] Savarese G., Becher P.M., Lund L.H., Seferovic P., Rosano G.M.C., Coats A.J.S. (2022). Global burden of heart failure: A comprehensive and updated review of epidemiology. Cardiovasc. Res..

[5] Sertkaya A., Birkenbach A., Berlind A., Eyraud J. (2014). Examination of Clinical Trial Costs and Barriers for Drug Development. U.S. Department of Health and Human Services. https://aspe.hhs.gov/sites/default/files/migrated_legacy_files/44516/rpt_erg.pdf.

[6] Dowden H., Munro J. (2019). Trends in clinical success rates and therapeutic focus. Nat. Rev. Drug Discov..

[7] Xue H., Li J., Xie H., Wang Y. (2018). Review of Drug Repositioning Approaches and Resources. Int. J. Biol. Sci..

[8] Hauser A.S., Attwood M.M., Rask-Andersen M., Schiöth H.B., Gloriam D.E. (2017). Trends in GPCR drug discovery: New agents, targets and indications. Nat. Rev. Drug Discov..

[9] McDonagh T.A., Metra M., Adamo M., Gardner R.S., Baumbach A., Böhm M., Burri H., Butler J., Čelutkienė J., Chioncel O. (2021). 2021 ESC Guidelines for the diagnosis and treatment of acute and chronic heart failure. Eur. Heart J..

[10] Wang J., Gareri C., Rockman H.A. (2018). G-Protein–Coupled Receptors in Heart Disease. Circ. Res..

[11] Tsuji Y. (2020). Transmembrane protein western blotting: Impact of sample preparation on detection of SLC11A2 (DMT1) and SLC40A1 (ferroportin). PLoS ONE.

[12] Corney D.C. (2013). RNA-seq Using Next Generation Sequencing. Mater. Methods.

[13] Taylor S.C., Laperriere G., Germain H. (2017). Droplet Digital PCR versus qPCR for gene expression analysis with low abundant targets: From variable nonsense to publication quality data. Sci. Rep..

[14] Onódi Z., Ruppert M., Kucsera D., Sayour A.A., Tóth V.E., Koncsos G., Novák J., Brenner G.B., Makkos A., Baranyai T. (2021). AIM2-driven inflammasome activation in heart failure. Cardiovasc. Res..

[15] Ruppert M., Lakatos B.K., Braun S., Tokodi M., Karime C., Oláh A., Sayour A.A., Hizoh I., Barta B.A., Merkely B. (2020). Longitudinal Strain Reflects Ventriculoarterial Coupling Rather Than Mere Contractility in Rat Models of Hemodynamic Overload–Induced Heart Failure. J. Am. Soc. Echocardiogr..

[16] Miyazaki T., Otani K., Chiba A., Nishimura H., Tokudome T., Takano-Watanabe H., Matsuo A., Ishikawa H., Shimamoto K., Fukui H. (2018). A New Secretory Peptide of Natriuretic Peptide Family, Osteocrin, Suppresses the Progression of Congestive Heart Failure after Myocardial Infarction. Circ. Res..

[17] Brattelid T., Qvigstad E., Moltzau L.R., Bekkevold S.V.S., Sandnes D.L., Birkeland J.A.K., Skomedal T., Osnes J.-B., Sjaastad I., Levy F.O. (2012). The Cardiac Ventricular 5-HT4 Receptor Is Functional in Late Foetal Development and Is Reactivated in Heart Failure. PLoS ONE.

[18] Yu Y., Lucitt M.B., Stubbe J., Cheng Y., Friis U.G., Hansen P.B., Jensen B.L., Smyth E.M., FitzGerald G.A. (2009). Prostaglandin F _2α_ elevates blood pressure and promotes atherosclerosis. Proc. Natl. Acad. Sci. USA.

[19] Wong S.L., Leung F.P., Lau C.W., Au C.L., Yung L.M., Yao X., Chen Z.-Y., Vanhoutte P.M., Gollasch M., Huang Y. (2009). Cyclooxygenase-2–Derived Prostaglandin F _2α_ Mediates Endothelium-Dependent Contractions in the Aortae of Hamsters with Increased Impact during Aging. Circ. Res..

[20] Oga T., Matsuoka T., Yao C., Nonomura K., Kitaoka S., Sakata D., Kita Y., Tanizawa K., Taguchi Y., Chin K. (2009). Prostaglandin F_2α_ receptor signaling facilitates bleomycin-induced pulmonary fibrosis independently of transforming growth factor-β. Nat. Med..

[21] Ding W.-Y., Ti Y., Wang J., Wang Z.-H., Xie G.-L., Shang Y.-Y., Tang M.-X., Zhang Y., Zhang W., Zhong M. (2012). Prostaglandin F2α facilitates collagen synthesis in cardiac fibroblasts via an F-prostanoid receptor/protein kinase C/Rho kinase pathway independent of transforming growth factor β1. Int. J. Biochem. Cell Biol..

[22] Wu Y., Cui C., Bi F.-F., Wu C.-Y., Li J.-R., Hou Y.-M., Jing Z.-H., Pan Q.-M., Cao M., Lv L.-F. (2022). Montelukast, cysteinyl leukotriene receptor 1 antagonist, inhibits cardiac fibrosis by activating APJ. Eur. J. Pharmacol..

[23] Tabula Muris Consortium, Overall Coordination, Logistical Coordination, Organ Collection and Processing, Library Preparation and Sequencing, Computational Data Analysis, Cell Type Annotation, Writing Group, Supplemental Text Writing Group, Principal Investigators (2018). Single-cell transcriptomics of 20 mouse organs creates a *Tabula Muris*. Nature.

[24] Schoger E., Bleckwedel F., Germena G., Rocha C., Tucholla P., Sobitov I., Möbius W., Sitte M., Lenz C., Samak M. (2023). Single-cell transcriptomics reveal extracellular vesicles secretion with a cardiomyocyte proteostasis signature during pathological remodeling. Commun. Biol..

[25] Onódi Z., Visnovitz T., Kiss B., Hambalkó S., Koncz A., Ágg B., Váradi B., Tóth V., Nagy R.N., Gergely T.G. (2021). Systematic transcriptomic and phenotypic characterization of human and murine cardiac myocyte cell lines and primary cardiomyocytes reveals serious limitations and low resemblances to adult cardiac phenotype. J. Mol. Cell. Cardiol..

[26] Fujimori K., Ueno T., Nagata N., Kashiwagi K., Aritake K., Amano F., Urade Y. (2010). Suppression of Adipocyte Differentiation by Aldo-keto Reductase 1B3 Acting as Prostaglandin F2α Synthase. J. Biol. Chem..

[27] Goupil E., Wisehart V., Khoury E., Zimmerman B., Jaffal S., Hébert T.E., Laporte S.A. (2012). Biasing the Prostaglandin F2α Receptor Responses toward EGFR-Dependent Transactivation of MAPK. Mol. Endocrinol..

[28] Harks E.G.A., Peters P.H.J., van Dongen J.L.J., van Zoelen E.J.J., Theuvenet A.P.R. (2005). Autocrine production of prostaglandin F_2α_enhances phenotypic transformation of normal rat kidney fibroblasts. Am. J. Physiol. Cell Physiol..

[29] Jelemenský M., Kovácsházi C., Ferenczyová K., Hofbauerová M., Kiss B., Pállinger É., Kittel Á., Sayour V.N., Görbe A., Pelyhe C. (2021). Helium Conditioning Increases Cardiac Fibroblast Migration Which Effect Is Not Propagated via Soluble Factors or Extracellular Vesicles. Int. J. Mol. Sci..

[30] Weber B.Y., Brenner G.B., Kiss B., Gergely T.G., Sayour N.V., Tian H., Makkos A., Görbe A., Ferdinandy P., Giricz Z. (2022). Rosiglitazone Does Not Show Major Hidden Cardiotoxicity in Models of Ischemia/Reperfusion but Abolishes Ischemic Preconditioning-Induced Antiarrhythmic Effects in Rats In Vivo. Pharmaceuticals.

[31] Brenner G.B., Makkos A., Nagy C.T., Onódi Z., Sayour N.V., Gergely T.G., Kiss B., Görbe A., Sághy É., Zádori Z.S. (2020). Hidden Cardiotoxicity of Rofecoxib Can be Revealed in Experimental Models of Ischemia/Reperfusion. Cells.

[32] Sayour N.V., Brenner G.B., Makkos A., Kiss B., Kovácsházi C., Gergely T.G., Aukrust S.G., Tian H., Zenkl V., Gömöri K. (2023). Cardioprotective efficacy of limb remote ischaemic preconditioning in rats: Discrepancy between a meta-analysis and a three-centre in vivo study. Cardiovasc. Res..

[33] Brenner G.B., Giricz Z., Garamvölgyi R., Makkos A., Onódi Z., Sayour N.V., Gergely T.G., Baranyai T., Petneházy Ö., Kőrösi D. (2021). Post-Myocardial Infarction Heart Failure in Closed-chest Coronary Occlusion/Reperfusion Model in Göttingen Minipigs and Landrace Pigs. J. Vis. Exp..

[34] Chaffin M., Papangeli I., Simonson B., Akkad A.-D., Hill M.C., Arduini A., Fleming S.J., Melanson M., Hayat S., Kost-Alimova M. (2022). Single-nucleus profiling of human dilated and hypertrophic cardiomyopathy. Nature.

[35] Zhang J., Gong Y., Yu Y. (2010). PG F2α Receptor: A Promising Therapeutic Target for Cardiovascular Disease. Front. Pharmacol..

[36] Beccacece L., Abondio P., Bini C., Pelotti S., Luiselli D. (2023). The Link between Prostanoids and Cardiovascular Diseases. Int. J. Mol. Sci..

[37] Rabinowitz B., Arad M., Elazar E., Klein R., Zahav Y.H. (1992). Epicardial versus endocardial "in mirror" changes in prostaglandin synthesis after short periods of ischemia and reperfusion. Eicosanoids.

[38] Yoshida M., Sagawa N., Itoh H., Yura S., Takemura M., Wada Y., Sato T., Ito A., Fujii S. (2002). Prostaglandin F2alpha, cytokines and cyclic mechanical stretch augment matrix metalloproteinase-1 secretion from cultured human uterine cervical fibroblast cells. Mol. Hum. Reprod..

[39] Almirza W., Dernison M., Peters P., van Zoelen E., Theuvenet A. (2008). Role of the prostanoid FP receptor in action potential generation and phenotypic transformation of NRK fibroblasts. Cell. Signal..

[40] Lai J., Jin H., Yang R., Winer J., Li W., Yen R., King K.L., Zeigler F., Ko A., Cheng J. (1996). Prostaglandin F2 alpha induces cardiac myocyte hypertrophy in vitro and cardiac growth in vivo. Am. J. Physiol. Circ. Physiol..

[41] Mallat Z., Philip I., Lebret M., Chatel D., Maclouf J., Tedgui A. (1998). Elevated Levels of 8-iso-Prostaglandin F _2α_ in Pericardial Fluid of Patients with Heart Failure. Circulation.

[42] Cohen D., Koh G.Y., Nikonova L.N., Porter J.G., Maack T. (1996). Molecular Determinants of the Clearance Function of Type C Receptors of Natriuretic Peptides. J. Biol. Chem..

[43] Nussenzveig D.R., Lewicki J.A., Maack T. (1990). Cellular mechanisms of the clearance function of type C receptors of atrial natriuretic factor. Perspect. Surg..

[44] Venkatesan B., Tumala A., Subramanian V., Vellaichamy E. (2016). Transient silencing of Npr3 gene expression improved the circulatory levels of atrial natriuretic peptides and attenuated β-adrenoceptor activation- induced cardiac hypertrophic growth in experimental rats. Eur. J. Pharmacol..

[45] Wong L.L., Wee A.S., Lim J.Y., Ng J.Y., Chong J.P., Liew O.W., Lilyanna S., Martinez E.C., Ackers-Johnson M.A., Vardy L.A. (2015). Natriuretic peptide receptor 3 (NPR3) is regulated by microRNA-100. J. Mol. Cell. Cardiol..

[46] Wang J., Tong K.S., Wong L.L., Liew O.-W., Raghuram D., Richards A.M., Chen Y.-T. (2018). MicroRNA-143 modulates the expression of Natriuretic Peptide Receptor 3 in cardiac cells. Sci. Rep..

[47] Sato T., Sato C., Kadowaki A., Watanabe H., Ho L., Ishida J., Yamaguchi T., Kimura A., Fukamizu A., Penninger J.M. (2017). ELABELA-APJ axis protects from pressure overload heart failure and angiotensin II-induced cardiac damage. Cardiovasc. Res..

[48] Azizi Y., Faghihi M., Imani A., Roghani M., Nazari A. (2013). Post-infarct treatment with [Pyr1]-apelin-13 reduces myocardial damage through reduction of oxidative injury and nitric oxide enhancement in the rat model of myocardial infarction. Peptides.

[49] Wang W., McKinnie S.M., Patel V.B., Haddad G., Wang Z., Zhabyeyev P., Das S.K., Basu R., McLean B., Kandalam V. (2013). Loss of Apelin Exacerbates Myocardial Infarction Adverse Remodeling and Ischemia-reperfusion Injury: Therapeutic Potential of Synthetic Apelin Analogues. J. Am. Heart Assoc..

[50] Hoxha M., Tedesco C.C., Quaglin S., Malaj V., Pustina L., Capra V., Evans J.F., Sala A., Rovati G.E. (2021). Montelukast Use Decreases Cardiovascular Events in Asthmatics. Front. Pharmacol..

[51] Ingelsson E., Yin L., Bäck M. (2012). Nationwide cohort study of the leukotriene receptor antagonist montelukast and incident or recurrent cardiovascular disease. J. Allergy Clin. Immunol..

[52] Sarszegi Z., Szabo D., Gaszner B., Konyi A., Reglodi D., Nemeth J., Lelesz B., Polgar B., Jungling A., Tamas A. (2018). Examination of Pituitary Adenylate Cyclase-Activating Polypeptide (PACAP) as a Potential Biomarker in Heart Failure Patients. J. Mol. Neurosci..

[53] Szabó D., Sárszegi Z., Polgár B., Sághy É., Reglődi D., Tóth T., Onódi Z., Leszek P., Varga Z.V., Helyes Z. (2022). PACAP-38 and PAC1 Receptor Alterations in Plasma and Cardiac Tissue Samples of Heart Failure Patients. Int. J. Mol. Sci..

[54] Packer M., McMurray J.J., Krum H., Kiowski W., Massie B.M., Caspi A., Pratt C.M., Petrie M.C., DeMets D., Kobrin I. (2017). Long-Term Effect of Endothelin Receptor Antagonism with Bosentan on the Morbidity and Mortality of Patients with Severe Chronic Heart Failure. JACC Heart Fail..

[55] Gao C., Ren S.V., Yu J., Baal U., Thai D., Lu J., Zeng C., Yan H., Wang Y. (2019). Glucagon Receptor Antagonism Ameliorates Progression of Heart Failure. JACC Basic Transl. Sci..

[56] Kolur V., Vastrad B., Vastrad C., Kotturshetti S., Tengli A. (2021). Identification of candidate biomarkers and therapeutic agents for heart failure by bioinformatics analysis. BMC Cardiovasc. Disord..

[57] Martin M. (2011). Cutadapt removes adapter sequences from high-throughput sequencing reads. EMBnet J..

[58] Williams C.R., Baccarella A., Parrish J.Z., Kim C.C. (2016). Trimming of sequence reads alters RNA-Seq gene expression estimates. BMC Bioinform..

[59] Ewels P., Magnusson M., Lundin S., Käller M. (2016). MultiQC: Summarize analysis results for multiple tools and samples in a single report. Bioinformatics.

[60] Kim D., Langmead B., Salzberg S.L. (2015). HISAT: A fast spliced aligner with low memory requirements. Nat. Methods.

[61] Liao Y., Smyth G.K., Shi W. (2014). featureCounts: An efficient general purpose program for assigning sequence reads to genomic features. Bioinformatics.

[62] Li H., Handsaker B., Wysoker A., Fennell T., Ruan J., Homer N., Marth G., Abecasis G., Durbin R., 1000 Genome Project Data Processing Subgroup (2009). The Sequence Alignment/Map format and SAMtools. Bioinformatics.

[63] Danecek P., Bonfield J.K., Liddle J., Marshall J., Ohan V., Pollard M.O., Whitwham A., Keane T., McCarthy S.A., Davies R.M. (2021). Twelve years of SAMtools and BCFtools. GigaScience.

[64] Love M.I., Huber W., Anders S. (2014). Moderated estimation of fold change and dispersion for RNA-seq data with DESeq2. Genome Biol..

[65] Benjamini Y., Hochberg Y. (1995). Controlling the False Discovery Rate: A Practical and Powerful Approach to Multiple Testing. J. R. Stat. Soc. Ser. B Methodol..

[66] Gorbe A., Giricz Z., Szunyog A., Csont T., Burley D.S., Baxter G.F., Ferdinandy P. (2010). Role of cGMP-PKG signaling in the protection of neonatal rat cardiac myocytes subjected to simulated ischemia/reoxygenation. Basic Res. Cardiol..

[67] Lanner J.T., Georgiou D.K., Joshi A.D., Hamilton S.L. (2010). Ryanodine Receptors: Structure, Expression, Molecular Details, and Function in Calcium Release. Cold Spring Harb. Perspect. Biol..

[68] Feng D., Nagy J.A., Pyne K., Dvorak H.F., Dvorak A.M. (2004). Ultrastructural Localization of Platelet Endothelial Cell Adhesion Molecule (PECAM-1, CD31) in Vascular Endothelium. J. Histochem. Cytochem..

[69] Tamiolakis D., Papadopoulos N., Sivridis E., Anastasiadis P., Karamanidis D., Romanidis C., Kotini A., Bounovas A., Simopoulos C. (2001). Expression of the intermediate filament vimentin and fibrillar proteins of the extracellular matrix related to embryonal heart development. Clin. Exp. Obstet. Gynecol..

[70] Lawson J.S., Syme H.M., Wheeler-Jones C.P.D., Elliott J. (2018). Characterisation of feline renal cortical fibroblast cultures and their transcriptional response to transforming growth factor β1. BMC Vet. Res..

[71] Lees-Miller J.P., Heeley D.H., Smillie L.B. (1987). An abundant and novel protein of 22 kDa (SM22) is widely distributed in smooth muscles. Purification from bovine aorta. Biochem. J..

[72] Greaves D.R., Gordon S. (2002). Macrophage-Specific Gene Expression: Current Paradigms and Future Challenges. Int. J. Hematol..

