# Supplementary material for: Droplet Digital PCR Is a Novel Screening Method Identifying Potential Cardiac G-Protein-Coupled Receptors as Candidate Pharmacological Targets in a Rat Model of Pressure-Overload-Induced Cardiac Dysfunction
Source: Int J Mol Sci. 2023 Sep 7;24(18):13826. doi: 10.3390/ijms241813826 (PMC10531061; doi:10.3390/ijms241813826)
Supplement: Supplementary file 1 [file ijms-24-13826-s001.zip › ijms-2541560-supplementary.pdf]

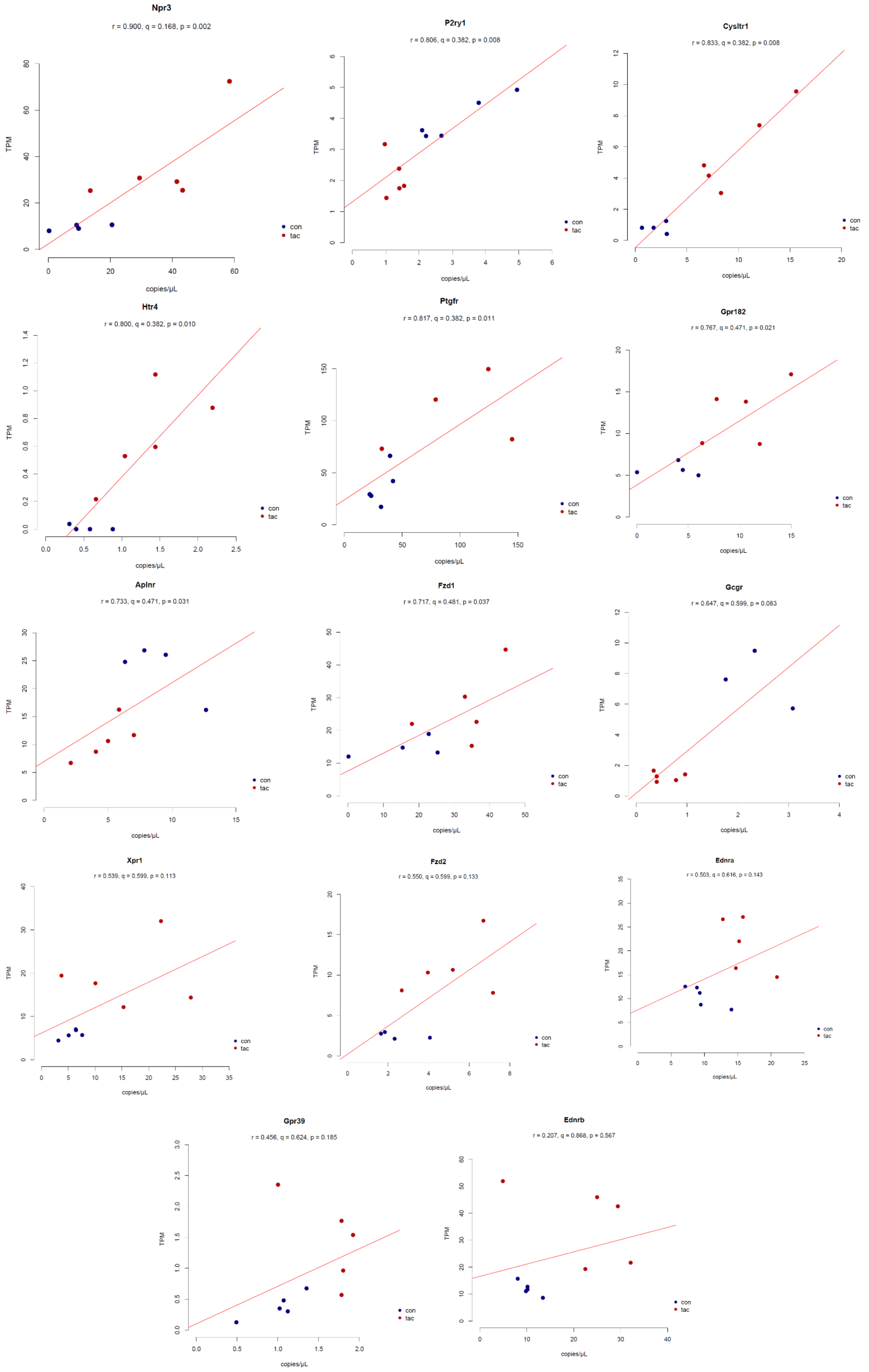

Npr3
r = 0.900, q = 0.168, p = 0.002
P2ry1
r = 0.806, q = 0.382, p = 0.008
Cysltr1
r = 0.833, q = 0.382, p = 0.008
Htr4
r = 0.800, q = 0.382, p = 0.010
Ptgfr
r = 0.817, q = 0.382, p = 0.011
Gpr182
r = 0.767, q = 0.471, p = 0.021
Aplnr
r = 0.733, q = 0.471, p = 0.031
Fzd1
r = 0.717, q = 0.481, p = 0.037
Gcgr
r = 0.647, q = 0.599, p = 0.083
Xpr1
r = 0.539, q = 0.599, p = 0.113
Fzd2
r = 0.550, q = 0.599, p = 0.133
Ednra
r = 0.503, q = 0.616, p = 0.143
Gpr39
r = 0.456, q = 0.624, p = 0.185
Ednrb
r = 0.207, q = 0.868, p = 0.567
TPM
copies/µL
con
tac

Figure S1. Correlations of expressions of genes encoding G–protein–coupled receptors in TAC vs. SHAM rat hearts measured by bulk RNA sequencing and droplet digital PCR. For each GPCR gene, measurements were obtained from n = 5 individual rat heart from both the SHAM (marked blue, termed as "con") and the TAC (marked red, termed as "red") groups. For correlation analyses, Pearsons rho (r) was calculated, and genes are ordered according to the significance of the correlation. Npr3: natriuretic peptide clearance receptor 3; P2ry1: purinergic receptor P2Y1; Cysltr1: cysteinyl leukotriene receptor 1; Htr4: 5-hydroxytryptamine receptor 4; Ptgfr: prostaglandin F2α receptor; Gpr182: G-protein coupled receptor 182; Aplnr: apelin receptor; Fzd1: frizzled receptor 1; Gcgr: glucagone receptor; Xpr1: xenotropic and polytropic retrovirus receptor 1; Fzd2: frizzled receptor 2; Ednra: endothelin-1 receptor type A; Gpr39: G-protein coupled receptor 39; Ednrb: endothelin-1 receptor type B.

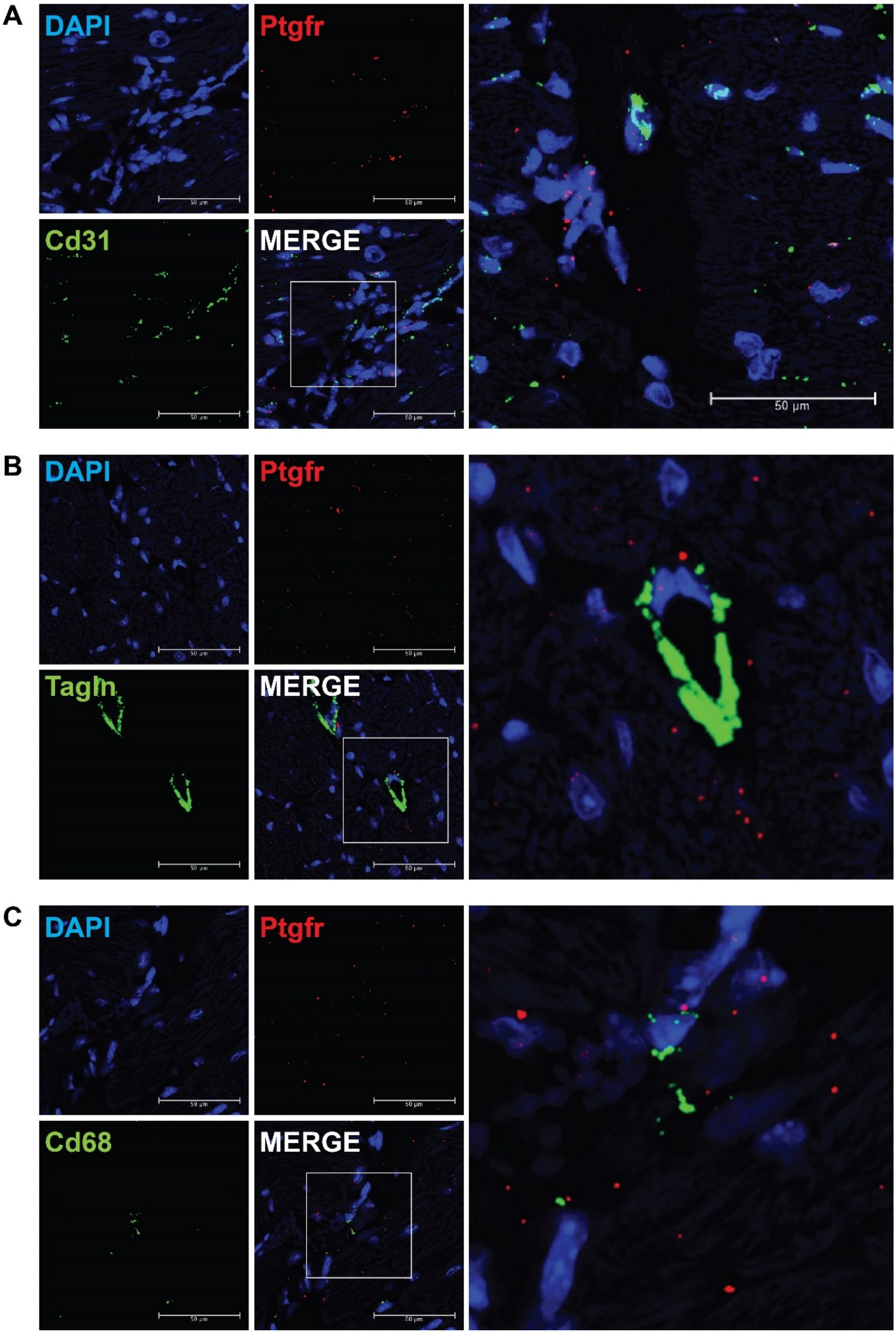

Figure S2. Prostaglandin $F_{2\alpha}$ receptor expression profile in murine hearts. (**A–C**) demonstrated representative RNA-Scope images of murine hearts showing *Ptgfr* expression on endothelial cells (CD31$^+$ cells), vascular smooth muscle cells (Tagln$^+$ cells), and immune cells (CD68$^+$ cells), respectively, with no technical replication or quantification.

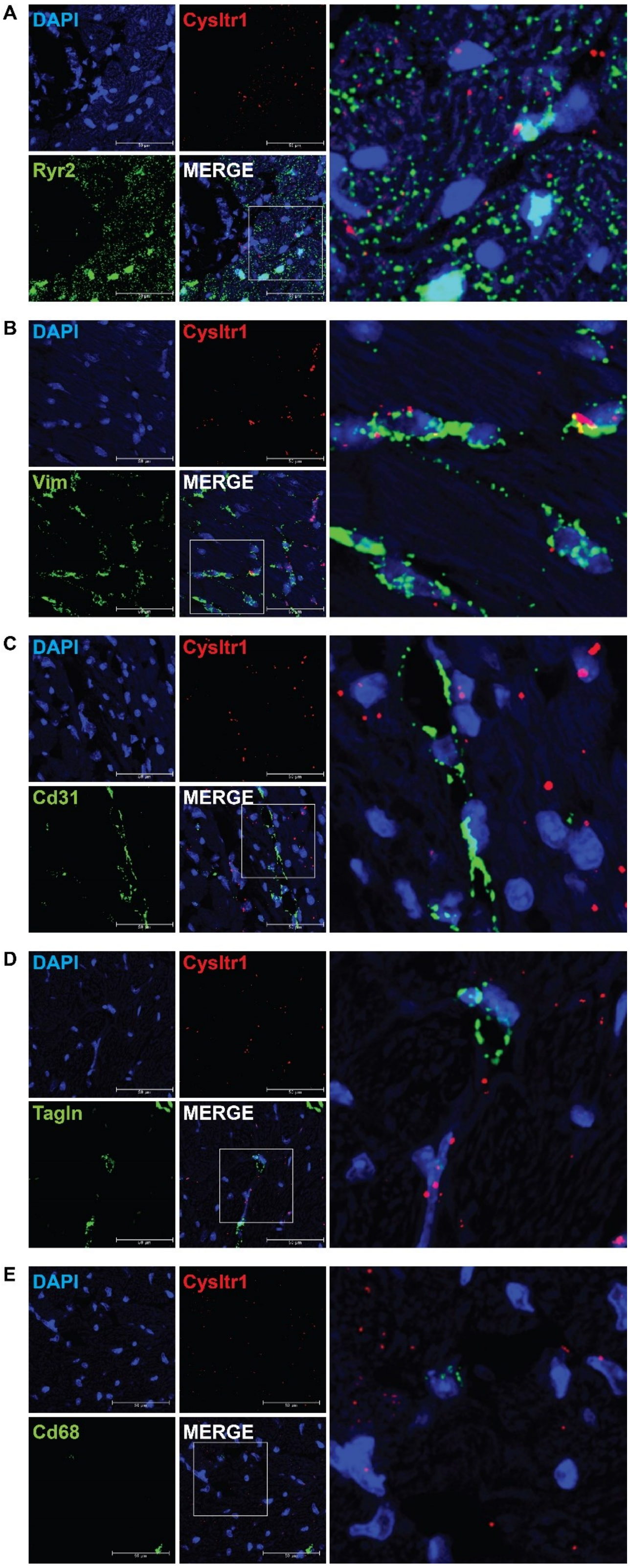

Figure S3. Cysteinyl leukotriene receptor 1 expression profile in murine hearts. (**A**–**E**) demonstrated representative RNA-Scope images of murine hearts showing *Cysltr1* expression on cardiomyocytes (Ryr2^{+} cells), cardiac fibroblasts (Vim^{+} cells), endothelial cells (CD31^{+} cells), vascular smooth muscle cells (Tagln^{+} cells), and immune cells (CD68^{+} cells), respectively, with no technical replication or quantification.

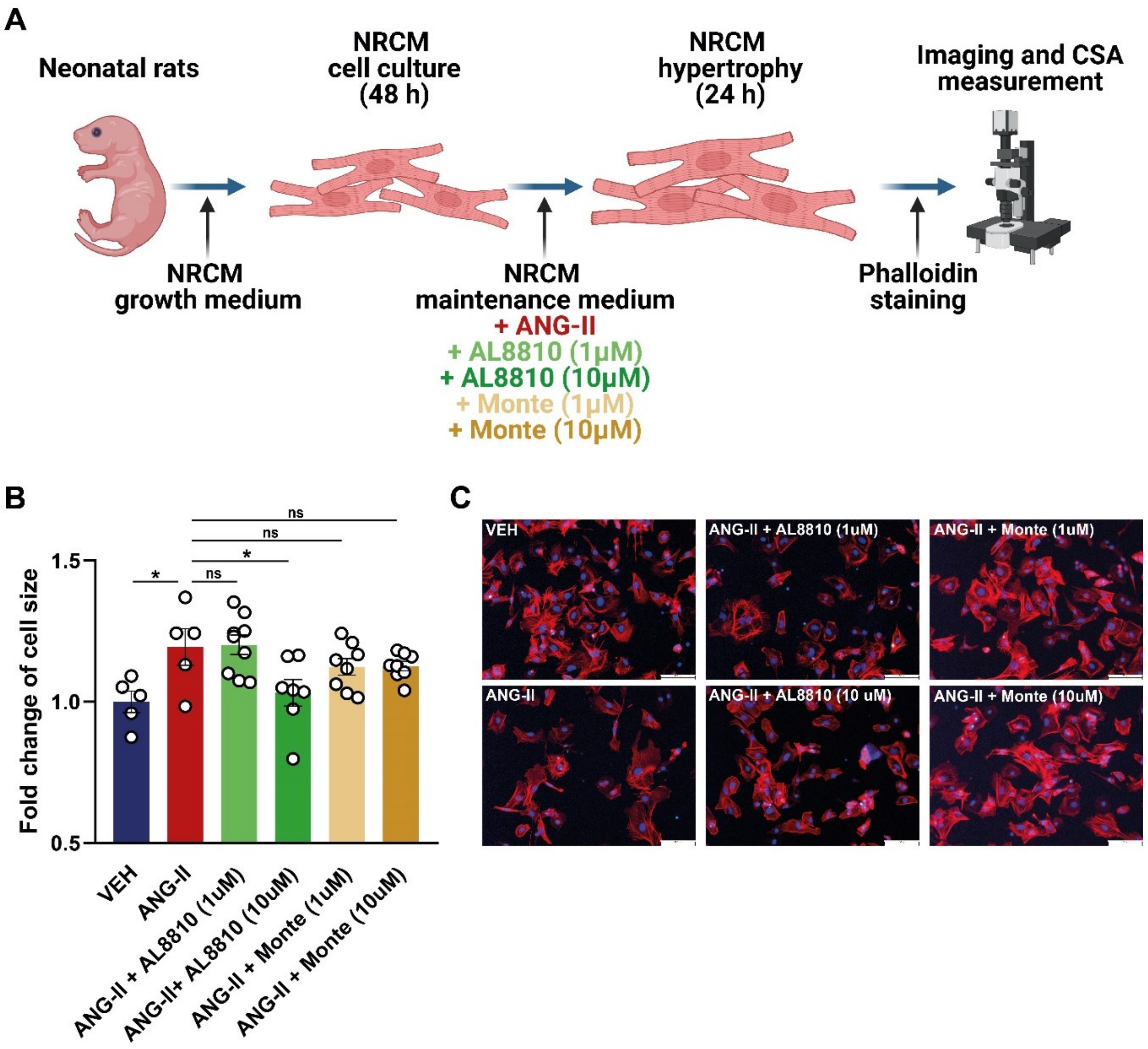

Figure S4. Effects of Prostaglandin $F_{2\alpha}$ receptor inhibition or Cysteinyl leukotriene receptor 1 inhibition on in vitro angiotensin-II-induced cardiomyocyte hypertrophy. (**A**) represents the experimental workflow of neonatal rat cardiomyocyte (NRCM) cell culturing, induction of hypertrophy by angiotensin-II (ANG-II), treatment with prostaglandin $F_{2\alpha}$ receptor (*Ptgfr*) antagonist AL8810, or with Cysteinyl leukotriene receptor 1 (*Cysltr1*) antagonist montelukast (Monte) in lower (1 μM) and higher (10 μM) doses, and measurement of cell surface area (CSA) after phalloidin staining. (**B**) shows significant increase in CSA of NRCM cells by ANG-II, which could be reverted by 10 μM of AL8810, but not with Monte; biological replicates of n = 5–9/group, *: $p < 0.05$ vs. ANG-II, One-way ANOVA, Dunnett's post hoc test, shown as mean ± SEM; ns: not significant. (**C**) shows representative images of phalloidin staining for each treatment groups.
